# Low-field and portable MRI technology: advancements and innovations

**DOI:** 10.1186/s41747-025-00638-2

**Published:** 2025-10-23

**Authors:** Dmitrij Kravchenko, Muhammad Taha Hagar, Milan Vecsey-Nagy, Ildiko Kabat, Anne Groteklaes, Julian A. Luetkens, Daniel Kuetting, Alexander Isaak, Tilman Emrich, Akos Varga-Szemes, Maria Vittoria Spampinato

**Affiliations:** 1 Department of Radiology and Radiological Science, Medical University of South Carolina, Charleston, SC USA; 2 Department of Diagnostic and Interventional Radiology, University Hospital Bonn, Bonn, Germany; 3 Quantitative Imaging Lab Bonn (QILaB), University Hospital Bonn, Bonn, Germany; 4 Department of Diagnostic and Interventional Radiology, Medical Centre, Faculty of Medicine, University of Freiburg, Freiburg im Breisgau, Germany; 5 Heart and Vascular Center, Semmelweis University, Budapest, Hungary; 6 Department of Neonatology and Pediatric Intensive Care, University Hospital Bonn, Bonn, Germany; 7 Department of Diagnostic and Interventional Radiology, University Medical Center of the Johannes Gutenberg-University, Mainz, Germany; 8 German Centre for Cardiovascular Research, Partner site Rhine-Main, Mainz, Germany

**Keywords:** Cost-benefit analysis, Magnetic resonance imaging, Point-of-care systems, Resource-limited settings, Signal-to-noise ratio

## Abstract

**Abstract:**

Recent advances in magnetic resonance imaging (MRI) hardware and software have renewed interest in low-field MRI, challenging the long-held notion that such systems are inherently inferior to high-field counterparts. Traditionally dismissed due to lower signal-to-noise ratios and reduced image quality, low-field MRI was primarily relegated to cost-sensitive or resource-limited settings. However, modern low-field systems now integrate advanced reconstruction algorithms, refined imaging techniques, and improved hardware design, significantly narrowing the performance gap. In some scenarios, these systems offer distinct advantages, such as reduced susceptibility artifacts and improved safety of metallic implants. Their portability, lower operational costs, and reduced infrastructure demands make them especially valuable in point-of-care, remote, or intraoperative environments. This review examines the physical principles of low-field MRI, traces its technological evolution, and evaluates its current and emerging clinical applications. By highlighting both its strengths and limitations, we aim to clarify the growing role of low-field MRI in contemporary diagnostic imaging and underscore its potential in expanding global access to high-quality radiological care.

**Relevance statement:**

Low-field and portable MRI systems offer a cost-effective, accessible, and safer imaging alternative that may expand diagnostic capabilities in underserved, point-of-care, and intraoperative settings, thereby improving global access to essential radiologic services.

**Key Points:**

- Advanced image reconstruction improves low-field MRI image quality and diagnostic utility.
- Reduced susceptibility artifacts enhance imaging near metallic hardware and air–tissue interfaces.
- Low-field systems enable cost-effective, portable imaging in constrained clinical environments.

**Graphical Abstract:**

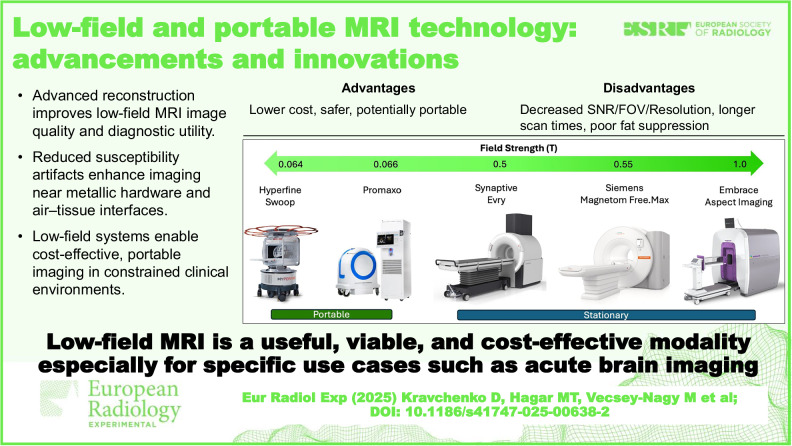

## Background

Magnetic resonance imaging (MRI) has become a cornerstone of diagnostic radiology, offering unparalleled soft tissue contrast and tissue characterization without the risks associated with ionizing radiation. Over the past few decades, MRI availability has expanded significantly, with nearly 40 million scans performed annually in the United States alone [1]. Of these, approximately 66% are conducted on 1.5-T scanners, 15–18% on 3.0-T systems, and the remainder on lower field strength devices [2]. Despite this widespread use, high-field MRI remains cost-prohibitive for many institutions, with an estimated 66% of the global population lacking access to MRI as of 2019 [3].

In the 1980s, low-field MRI systems—defined broadly as those operating at field strengths below 1.5-T—dominated the market. However, they fell out of favor due to lower spatial resolution and reduced signal-to-noise ratio (SNR) compared to high-field systems [4]. Historically, increasing static magnetic field strength was the primary method to improve SNR, while increasing coil density enhanced spatial resolution [5]. Yet, recent advances in magnet design, hardware miniaturization, artificial intelligence (AI), and image reconstruction techniques have reignited interest in low-field MRI [6]. Importantly, new insights have shown that static field strength is not the sole determinant of image quality; optimized hardware and software can significantly improve SNR, even at lower field strengths [7]. These innovations, coupled with specific clinical use cases, suggest a renewed role for low-field MRI in modern imaging. One of its greatest advantages is portability, enabling point-of-care and intraoperative imaging, as well as use in challenging environments such as intensive care units [8].

This review explores the current state of low-field MRI technology, its clinical applications, and operational considerations. We will focus on key areas where low-field systems show particular promise: acute brain imaging, neonatal and pediatric imaging, musculoskeletal applications, and interventional MRI.

## Low-field and high-field MRI systems

While a detailed discussion of MRI physics is beyond the scope of this review, a simplified overview of the core principles is useful to establish a common foundation for readers. Signal generation in MRI can be broken down into three key steps.

First, the static magnetic field (*B*_0_), measured in T, aligns the nuclear spins of hydrogen protons—primarily from water and fat—either parallel or antiparallel to the magnetic field. Although the vast majority of spins are equally distributed between these two states, a small excess aligns parallel to *B*₀, creating a net magnetization vector (*M*_0_). Second, a brief pulse of radiofrequency (RF) energy at the resonance frequency of hydrogen excites these protons, tipping *M*_0_ into the transverse plane. When the RF pulse ceases, the protons begin to relax back toward equilibrium. Two relaxation processes are of primary interest: T1 relaxation, which represents recovery of longitudinal magnetization along the *B*_0_ axis, and T2 relaxation, which describes the loss of coherence (dephasing) in the transverse plane. Third, as protons relax, they induce oscillating magnetic fields that generate a voltage in the receiver coils. This signal is spatially encoded using phase- and frequency-encoding gradients and stored in a domain known as *k*-space. A Fourier transform is subsequently applied to convert this raw data into the anatomical images we interpret clinically. Characteristics of the magnet, of the RF coils, and of the gradient system contribute to the image quality provided by these machines.

Among the various factors that influence image quality, SNR is arguably the most important. SNR is directly proportional to the strength and homogeneity of the static magnetic field; higher field strengths generally produce higher SNR, resulting in improved spatial resolution and image clarity. However, as subsequent sections will show, recent innovations have begun to close the performance gap between low-field and high-field MRI systems through optimization of both hardware and software.

### Hardware and software

The earliest MRI scanners employed resistive magnets, which were inexpensive and relatively easy to manufacture. However, they were limited to a maximum field strength of approximately 0.28 T and required substantial power to operate. Permanent magnets emerged as an alternative, offering passive operation without electrical current and enabling open scanner designs. Nevertheless, they were constrained to a maximum field strength of 0.4 T, were sensitive to temperature fluctuations, lacked dynamic shimming capabilities, and offered limited gradient performance. The development of superconducting magnets—now standard in conventional 1.5-T, 3.0-T, and higher-field MRI systems—enabled stable field strengths and superior image quality. However, these systems are costly, require extensive infrastructure, are extremely heavy, and depend on liquid helium for cryogenic cooling [9].

### Magnet design and RF coils

To address the limitations of early low-field systems—such as excessive size, weight, energy consumption, low *B*_0_ homogeneity, and poor magnetic shielding—modern designs have incorporated significant engineering innovations. Many contemporary low-field systems now utilize compact superconducting magnets (*e.g*., Siemens MAGNETOM Free.Max, see below) or high-performance permanent magnets (*e.g*., Hyperfine Swoop, see below), which do not require active cooling or substantial electricity.

Despite differences in technology, most modern low-field scanners share key features: smaller physical footprint, reduced power demands, and sufficient *B*_0_ homogeneity for clinical imaging. These design improvements make them viable for point-of-care and mobile applications without sacrificing diagnostic capability. RF coils are integral to both transmitting excitation pulses and receiving the emitted magnetic resonance signal. Due to inherently lower SNR at reduced field strengths, coil optimization has been critical. This has been achieved through several innovations. Superconducting RF coils designed for operation at high- or low-temperatures, minimize resistance and thermal noise, improving SNR [10, 11]. Multimodal surface coils leverage multiple resonant modes to increase RF field efficiency, further enhancing image quality [12].

## Benefits of low-field and portable MRI systems

### Accessibility

The cost of MRI ownership is typically divided into three major components: hardware acquisition, transportation, and installation, and ongoing maintenance. As of 2015, the capital cost of an MRI system was estimated to approach $1 million USD per tesla of field strength, making high-field systems largely inaccessible for low-income countries and underserved rural areas [13]. More recent estimates suggest that a 0.55-T system may cost only 40–50% as much as a standard 1.5-T scanner [14]. Furthermore, due to their lower weight and reduced electromagnetic shielding requirements, low-field systems can reduce installation and transport costs by up to 70%. These systems often eliminate the need for reinforced flooring, copper shielding, and dedicated heating, ventilation, and air conditioning systems. Maintenance costs can also be significantly lower—up to 45% less—particularly for low-field scanners that do not require a quench pipe or cryogenic cooling. Additional savings may be realized through smaller room size requirements and reduced energy consumption. Together, these factors make low-field MRI a cost-effective and viable solution for resource-limited healthcare settings.

### Portability

High infrastructure demands and service costs have long prevented MRI from reaching remote or low-resource environments [15]. While mobile MRI units are available from major vendors, they are typically expensive and logistically challenging [16]. Recent innovations have enabled true portability. The Hyperfine Swoop was the first Food and Drug Administration (FDA)-cleared portable MRI system, allowing imaging to be brought directly to the patient. Deoni et al demonstrated proof-of-concept for home-based MRI by integrating a 0.064-T Hyperfine Swoop system into a standard consumer van, enabling in-home neuroimaging [17]. Roberts et al demonstrated the technical feasibility of performing low-field portable MRI scans during active ambulance transport using a telemedicine-equipped mobile stroke unit, successfully acquiring diagnostic-quality images of a phantom and human volunteer [18].

### Patient safety and comfort

Low-field MRI offers potential safety benefits by reducing the specific absorption rate (SAR) and magnetic forces on ferromagnetic materials, thereby decreasing the risk of device dislodgement or tissue heating in patients with implants [19]. However, safety is not absolute. Sanpitak et al demonstrated in phantom models that some localized heating at 0.55 T could exceed that observed at 1.5 T, cautioning against blanket assumptions of safety across all low-field systems [20]. Nonetheless, artifact reduction around metallic implants is a well-documented advantage of low-field MRI due to lower susceptibility effects [8, 21–23]. This makes low-field imaging particularly suitable for postoperative or orthopedic imaging. In terms of patient experience, low-field systems tend to be quieter and often feature larger bore sizes or open configurations, reducing claustrophobia and acoustic discomfort. These features have been cited as leading causes of improved patient tolerance and reduced scan termination rates [24, 25]. In studies of patient preference, comfort, and reduced noise were consistently ranked as major benefits of low-field MRI systems [24].

## Clinical applications

Although low-field MRI systems have become less common in high-income countries, they remain vital diagnostic tools in many low-resource settings. For instance, in Nigeria, low-field scanners account for approximately 77.6% of all installed MRI units [26]. While some modern low-field platforms aim to match or replace the functionality of standard 1.5-T and 3.0-T systems, others are designed for specific niche applications—such as portable acute neuroimaging or point-of-care diagnostics. Interestingly, early studies comparing diagnostic performance at different field strengths found no significant differences in several clinical applications. For instance, in neurological and hepatic imaging, low-field systems performed comparably to higher-field counterparts [27–30]. One notable study of 221 patients with multiple sclerosis demonstrated no difference in diagnostic accuracy between 0.5-T and 1.5-T scanners, with both yielding an area under the curve of 0.96 (95% confidence interval [CI]: 0.93–0.99) for lesion detection [29]. A summary of the advantages and limitations of low-field MRI is provided in Table 1.

**Table 1:** Potential advantages and disadvantages of low-field strength compared to high-field strength MRI systems

Advantages	Implications
Mobility	Point of care imaging, including intensive care and bedside imaging
	Pediatric imaging
	Intraoperative imaging
Safety	Decreased chance of metallic projectiles
	Decreased risk of device interactions
Potential open bore design	Decreased patient claustrophobia
Lower manufacturing costs	Increased accessibility for lower-income facilities
Lower power consumption	Decreased upkeep costs
Lower SAR	Lower heat absorption in metallic implants and device heating
	Fewer susceptibility artifacts

Disadvantages	Implications
Lower SNR	Reduced image quality
Decreased spatial resolution	Reduced image quality
Smaller field of view	Reduced image quality
Lower measurable chemical shift effects	Need for sequence optimization
Increased scan times	Higher chance of movement or breathing artifacts
Different T1 relaxation times	Reduced inter-field-strength comparability

Given the heterogeneity in field strength, magnet type, portability, and software capabilities, clinical performance varies widely across systems. Consequently, rather than generalizing, this review discusses the clinical utility of low-field MRI in the context of specific systems and targeted use cases. Particular focus is given to key applications in neurology, musculoskeletal imaging, and pediatrics, while acknowledging that additional areas, such as whole-body and functional imaging and cardiac imaging, are active areas of ongoing research and development.

### Neuroimaging

Neurological indications are among the most frequent reasons for MRI referrals, particularly in the evaluation of acute ischemic stroke, traumatic brain injury, multiple sclerosis, and hydrocephalus. While computed tomography remains the dominant modality in emergency settings due to speed and cost, it lacks the sensitivity of MRI for early ischemic changes and soft tissue differentiation [31]. Historically, low-field MRI was limited by prolonged acquisition times and inadequate resolution. For example, early 0.2-T diffusion-weighted imaging (DWI) systems required up to 52 min for 18 slices, rendering them impractical for acute settings [32]. However, recent low-field platforms, through the use of deep learning (DL), have demonstrated marked improvements in scan speed, image quality, and diagnostic performance, for example, improving acquisition times for T1- and T2- weighted images to 8.6 min and 11.2 min, respectively [33]. Studies now suggest that low-field neuroimaging, particularly with optimized protocols and AI-enhanced reconstruction, can approach the diagnostic accuracy of higher-field systems across a range of pathologies, such as stroke, hydrocephalus, intracranial hemorrhage, brain tumors, and multiple sclerosis [19].

### Musculoskeletal imaging

MRI plays a central role in evaluating joints, ligaments, tendons, and soft tissues, especially in sports medicine, orthopedics, and rheumatology. While 1.5-T and 3.0-T scanners remain standard in most clinical environments, low-field MRI is increasingly being adopted in outpatient clinics and ambulatory care settings due to its cost-efficiency, portability, and reduced infrastructure requirements [34]. Dedicated extremity scanners and compact systems have shown acceptable image quality for evaluating musculoskeletal pathology, particularly when combined with high-performance RF coils and advanced reconstruction methods [7, 35].

### Neonatal and pediatric imaging

MRI offers significant advantages in pediatric imaging due to its lack of ionizing radiation. However, the transport of critically ill neonates to conventional MRI suites presents substantial risks. Low-field MRI, with its smaller footprint, reduced acoustic noise, and enhanced safety profile, is well-suited for use in neonatal intensive care units and bedside environments. While cranial ultrasound remains common for evaluating neonatal brain pathology, low-field MRI is increasingly being used to assess conditions such as hypoxic-ischemic injury, intraventricular hemorrhage, and structural malformations [36–38].

### Cardiac imaging

The number of cardiac MRI examinations has been growing steadily over the last few years and is expected to continue on that trajectory [39]. Low-field MRI offers several theoretical and practical benefits for cardiac imaging. Lower field strengths result in reduced susceptibility artifacts and lower SAR, improving safety and diagnostic confidence in patients with cardiac implantable electronic devices. Additionally, low-field systems reduce off-resonance artifacts in balanced steady-state free precession sequences, which are commonly used for high-contrast cine imaging. As acquisition techniques and coil designs evolve, low-field systems may provide a viable and cost-effective alternative for routine cardiac magnetic resonance, particularly in resource-limited or implant-heavy populations [40, 41].

## Currently available low-field MRI machines

**Fig. 1:**
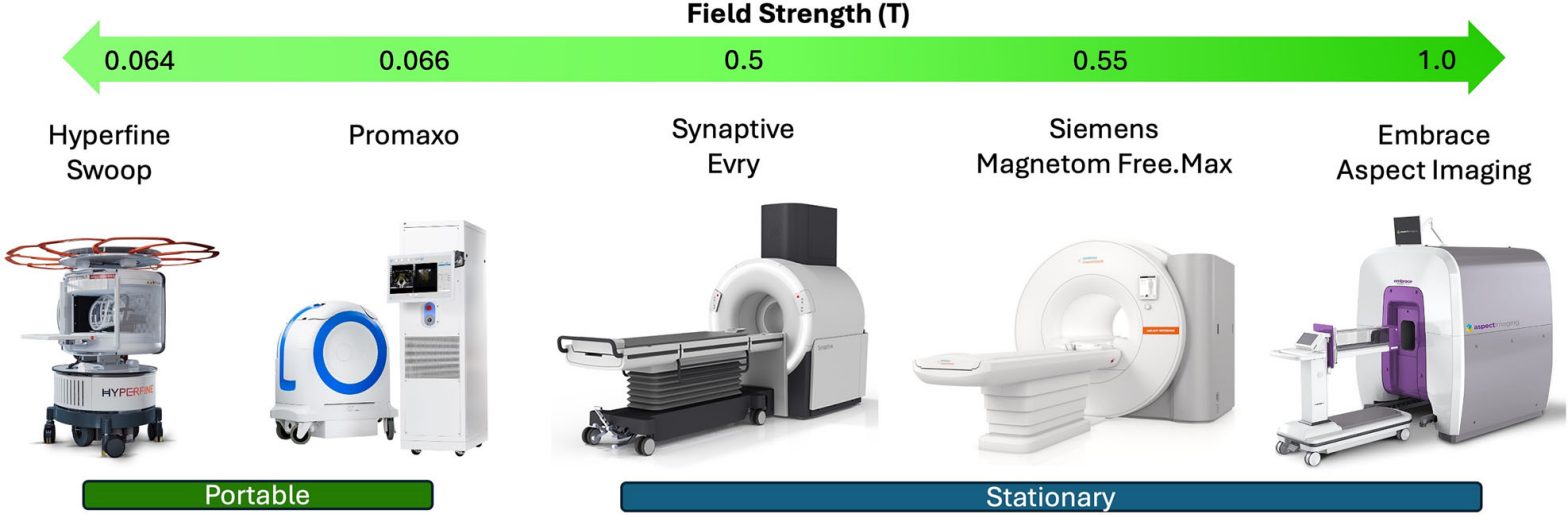
Selection of some currently available low-field MRI machines organized by field strength measured in Tesla (T) and by their mobility. Images courtesy of the respective vendors and Arnold et al with Wiley publishing under the Creative Commons CC-BY license [6]

While the more expensive 1.5-T and 3.0-T market is dominated by a limited number of vendors, the more cost-effective low-field MRI market offers a wider range of vendors and field strengths. The following list of systems is not exhaustive but represents an example of FDA-cleared machines currently available for purchase, as summarized in Table 2 and visually represented in Fig. 1.

**Table 2:** Selection of the current FDA-cleared low-field MRI systems

Manufacturer	Model	Field strength (T)	Use case
Aspect Imaging	Embrace	1.0-T	Point-of-care neonatal brain imaging
Siemens Healthineers	MAGNETOM Free.Max	0.55-T	Point-of-care-brain, neck, spine, abdomen, and joint imaging
Synaptive	Evry	0.5-T	Point-of-care brain imaging
Promaxo	−	0.066-T	Mobile MRI-guided prostate biopsies
Hyperfine	Swoop	0.064-T	Mobile brain imaging

### 1.0-T embrace

The 1.0-T Embrace (Aspect Imaging), point-of-care, self-shielded MRI system received FDA clearance in 2017 and is primarily used for brain imaging on neonatal intensive care units [42]. In a study of 207 brain MRIs read by pediatric neuroradiologists, this 1.0-T system demonstrated comparable performance to 3.0-T systems, with only one case showing discordant findings [43]. Notable findings assessed in the study included hemorrhage, stroke, tumors, and polymicrogyria, detected on T1-weighted fast spin-echo, T1-weighted three-dimensional (3D) gradient-echo, T2-weighted fast spin-echo, and DWI. In another study by Berson et al, 60 neonates were examined using this 1.0-T system and compared to transcranial ultrasound (*n* = 46) and to 3.0-T MRI (*n* = 3), with one scan terminated due to motion [36]. The authors noted two cases of findings visualized on the 3.0-T system that were not detected on the 1.0-T system, including microhemorrhage and small intraventricular hemorrhage. These findings suggest that while 1.0-T brain imaging generally offers concordance with 3.0-T, it may miss smaller abnormalities.

### 0.55-T MAGNETOM Free.Max

The MAGNETOM Free.Max (Siemens Healthineers) features an 80 cm bore and supports up to 220 kg (485 lbs) with a compact design, occupying less than 25 m². The machine utilizes a cryogenic subsystem housed in a sealed-for-life chassis, which requires only 0.7 L of liquid helium, compared to the 1,500 L typically required by traditional machines. Additionally, it facilitates interventional MRI procedures. Brain MRI is one of the most common use cases for low-field systems, and sufficient resolution and contrast are crucial for keeping these systems competitive with higher-field counterparts. A study by Osmanodja et al demonstrated that radiologists reading 0.55-T time-of-flight images detected all aneurysms seen on 1.5-T/3.0-T acquisitions [44]. Additionally, aneurysm size on the 0.55-T system was comparable to invasive angiography (3.61 ± 1.8 mm *versus* 3.47 ± 1.6 mm, *p* = 0.178). Another study showed no significant differences between 0.55-T and 1.5-T systems in diagnostic confidence or lesion conspicuity for vestibular schwannomas using T1- and T2-weighted imaging (*p* = 0.60–0.73) [45]. Acute stroke imaging is another critical application for low-field MRI systems. Rusche et al found that while noise on DWI and apparent diffusion coefficient sequences was scored higher on the 0.55-T system compared to the 1.5-T system (*p* < 0.026), all other sequences performed better on the 1.5-T system (*p* < 0.029). However, specificity (100%) and sensitivity (92.9%) for acute infarctions were identical between the two scanners [46].

Abdominal imaging has been proven to be feasible on low-field systems. Ramachandran et al produced diagnostic-quality abdominal MRI on a 0.55-T system, although longer acquisition times were required compared to higher-field systems. The study indicated that customized sequences, which enhanced SNR and reduced artifacts, allowed for adequate clinical reading in all patients (*n* = 52) by one reader and in 46 cases by the second reader. While 0.55-T scans showed lower image quality in certain gradient-echo sequences, they performed better in DWI. The average scan time at 0.55-T was significantly longer (54 ± 10 *versus* 36 ± 11 min, *p* < 0.001) [47].

With ever-increasing cardiac MRI studies being performed, low-field cardiac imaging is predestined to become an interesting focal point. Initial cardiac imaging feasibility studies using 0.55-T showed comparable volumetric results compared to 1.5-T for left ventricular end-diastolic volume (*p* = 0.77, bias = 0.40 mL, correlation coefficient = 0.99) and right ventricular end-diastolic volume (*p* = 0.17, bias = -1.6 mL, correlation coefficient = 0.98) [48]. Segeroth et al also reported an excellent mean correlation of *r* = 0.98 (95% CI: 0.97–0.98) for left and right ventricle volumetry [49]. Preliminary results for myocardial infarction detection on 0.55- and 1.5-T systems showed a 100% sensitivity in a cohort of 22 patients [50]. Additionally, strain measurements in phantom models for global longitudinal and circumferential strain showed similar results between the 0.55-T and 1.5-T systems (-19.4% ± 1.1 *versus* -18.7 ± 1.4%, *p* > 0.10) [51].

**Fig. 2:**
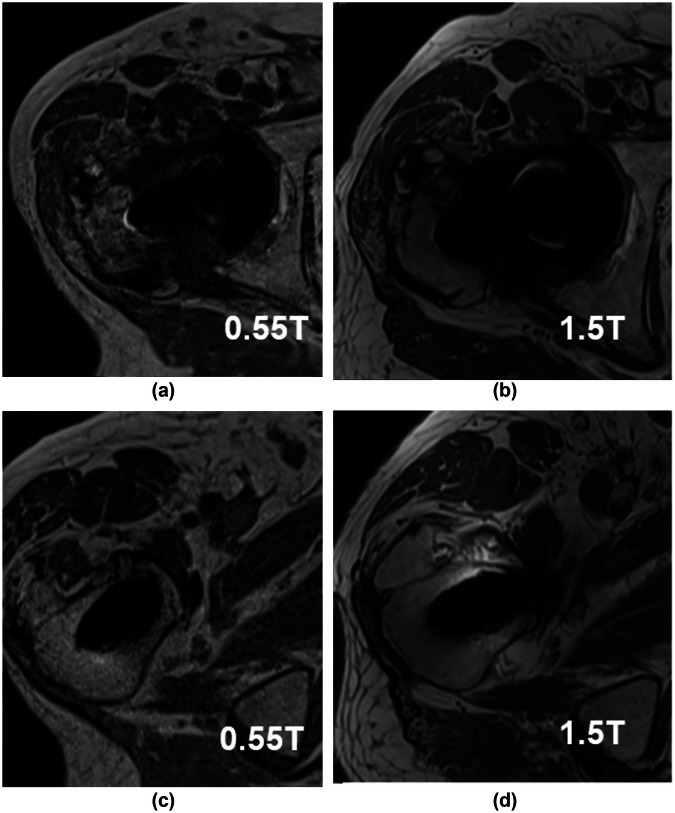
Comparison of artifacts at the level of the acetabulum (**a**, **b**) and proximal stem (**c**, **d**) at 0.55 and 1.5-T in the same patient on T1-weighted imaging. Reprinted with permission from Breit et al and Elsevier under the Creative Commons CC-BY license [53]

Musculoskeletal imaging with 0.55-T systems demonstrated that DL algorithms can help improve image quality to match that of 3.0-T systems. In a study by Donners et al, 0.55-T scans coupled with DL techniques were comparable to 3.0-T sequences for identifying and grading of high-grade cartilage and meniscal lesions, with good agreement (intraclass correlation coefficient [ICC] > 0.76), although agreement for low-grade cartilage lesions (ICC = 0.77) and meniscal lesions (ICC = 0.49) was somewhat lower [52]. Breit et al furthermore demonstrated less severe metal artifacts in patients with hip arthroplasty (Fig. 2) [53].

### 0.5-T Evry

The Evry 0.5-T MRI system (Synaptive Medical), FDA-approved in 2020, is designed for head and neck imaging in both adults and pediatric patients [54]. Like the other low-field systems, the Evry system can be installed outside traditional MRI suites. A study by Chaga et al found that this capability led to a decrease in MRI-to-treatment time from an average of 12 days (range 9–25 days) to 7 days (range 6–10 days, *p* < 0.001) and a reduction in consult-to-MRI time from 15 days (range 7–26 days) to 6 days (range 0–14 days, *p* = 0.004) for patients undergoing stereotactic radiosurgery [54]. Preliminary findings demonstrated adequate tissue contrast, lower SAR, and feasibility for DWI [55–57].

### 0.066-T Promaxo

The 0.066-T Promaxo MRI system (Promamxo) is an interventional device targeted at urologists for prostate imaging and biopsies. The system’s compact design allows installation in office settings, similar to ultrasound-guided biopsy systems. Sze et al compared the results of prostate biopsies in 39 men using Promaxo-guided transperineal biopsies and 12-core systematic transrectal ultrasound-guided biopsies [58]. The low-field MRI detected 74.3% of cancers, 53.8% of which were clinically significant prostate cancers, while ultrasound-guided biopsy detected 42.5% (*p* = 0.21). Chiragzada et al reported similar findings, with MRI detecting nine cases of prostate cancer missed by ultrasound and upgrading three cases undergraded by ultrasound, highlighting the advantages of point-of-care MRI for prostate cancer detection [59].

### 0.064-T Swoop

**Fig. 3:**
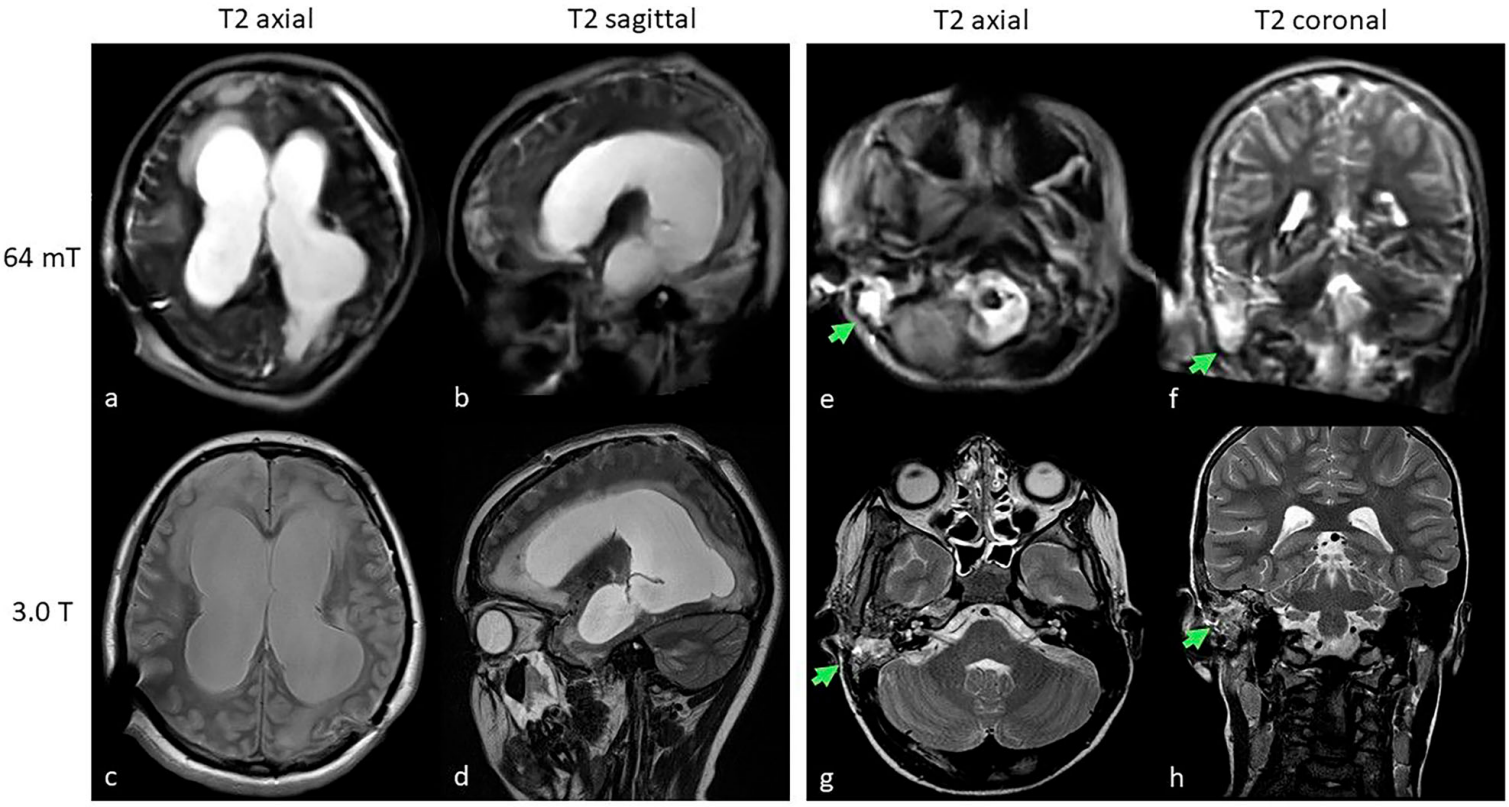
Utility of T2-weighted low-field MRI at 0.064-T (top row) acquired axial, sagittal, and coronal reconstructions in two different patients (Patient 1, **a**–**d**; Patient 2, **e**–**h**) compared to standard of care 3.0-T MRI (bottom row). Hydrocephalus was readily visualized on low-field MRI (**a**, **b**; dilated hyperintense ventricles) and confirmed on 3.0-T (**c**, **d**) in a 14-year-old female patient. Patient 2 (8-year-old male) demonstrated T2 hyperintensities of the right mastoid cells (arrows) on low-field (**e**, **f**) and confirmed on high-field MRI on the same day (**g**, **h**). While image quality on the 0.064-T Hyperfine Swoop® is not as sharp as on the high-field system, it was sufficient to provide a working diagnosis and initiate further testing in both cases

**Fig. 4:**
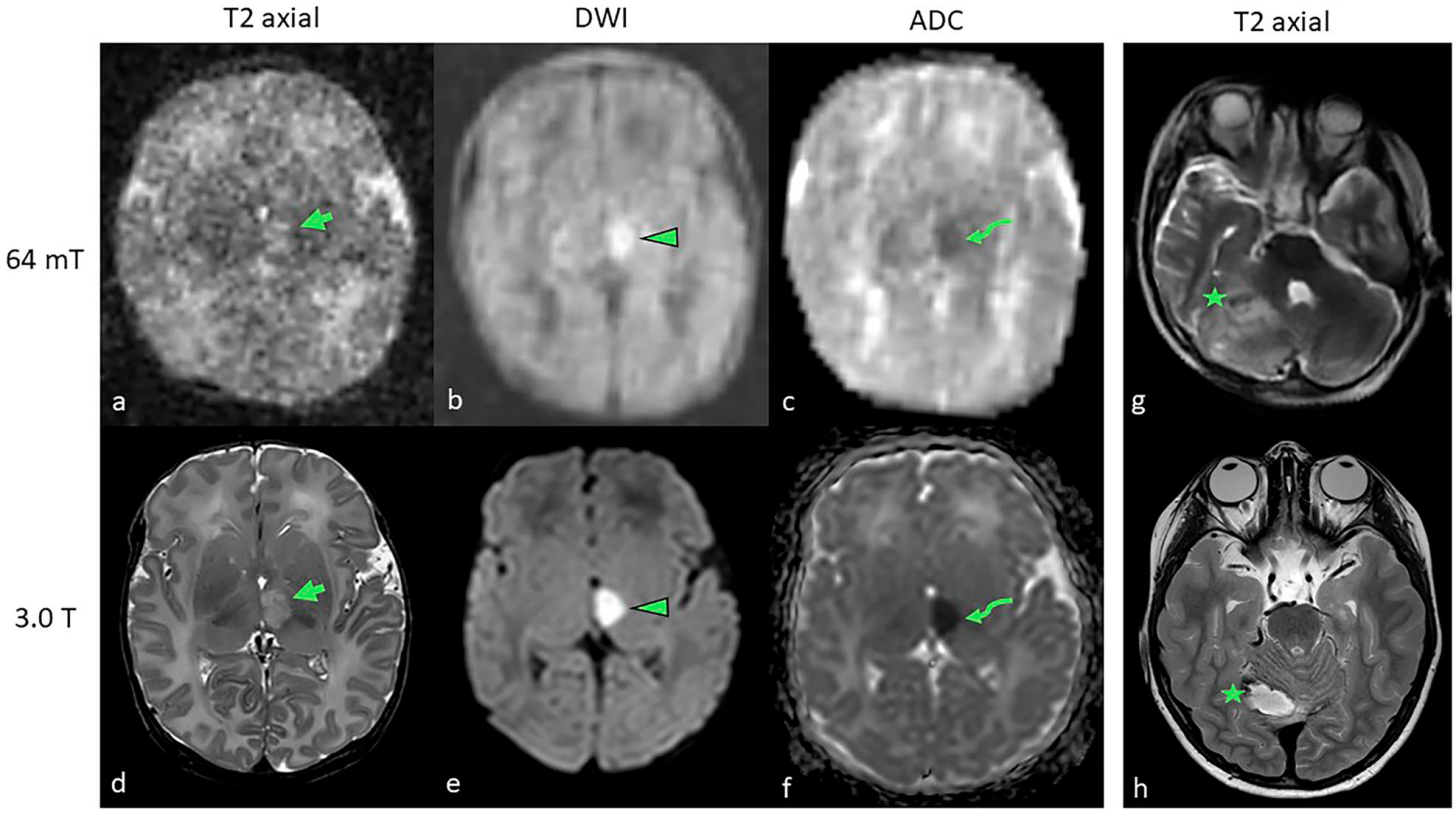
Low-field MRI at 0.064-T (top row) in two different patients (Patient 1, **a**–**f**, Patient 2, **g**, **h**) compared to 3.0-T (bottom row). Patient 1 (9-month-old male) demonstrates focal T2 hyperintense changes along the left thalamus (arrows) on both low-field (**a**) and high-field (**d**) images, as well as on diffusion-weighted images (DWI, **b** and **e**, respectively) with correlates on the apparent diffusion coefficient (ADC, **c** and **f**, respectively). Patient 2 (5-year-old male) shows a focal T2 hyperintense lesion with mass effect (star) between the brain stem and the right cerebellum as seen on the low-field (**g**) and confirmed on the high-field (**h**) sequences. The mass turned out to be an arteriovenous malformation with subarachnoid hemorrhage and edema. The patient required sedation for the high-field scan, while the low-field examination transpired without sedation within 2.2 min in the presence of a parent

The Swoop (Hyperfine Inc.), like the Promaxo system, is an ultra-low-field MRI system operating at 0.064-T (64-mT) and is designed for brain-only imaging. This fully portable system offers quieter operation and lower SARs, improving patient safety. Initial studies have demonstrated its potential when compared to 1.5-T and 3.0-T systems. A study evaluating multiple sclerosis lesions in 36 patients showed that the 64-mT system was noninferior to the 3.0-T system, identifying 94% of white matter lesions confirmed at 3.0-T [60]. Although smaller lesions were more difficult to identify, lesion volume estimates from 64-mT scans were strongly correlated with those from 3.0-T scans (*r* = 0.89, *p* < 0.001), though a systematic bias was noted. Lesion size was a key factor in detection accuracy, with super-resolution imaging techniques enhancing spatial detail in low-field systems. Donnay et al demonstrated that sparse sampling super-resolution could improve image quality, with expert neurologists scoring under-sampled 64-mT MRI images similarly to standard acquisitions [61]. Yuen et al found that stroke volumes on fluid-attenuated inversion recovery and DWI from the 0.064-T bedside MRI correlated well with 1.5-T systems (fluid-attenuated inversion recovery, ICC = 0.989, 95% CI: [0.976–0.995], *p* < 0.001; DWI, ICC = 0.940, 95% CI: [0.871–0.972], *p* < 0.001) [62]. Out of 50 patients, 45 (90%) were correctly identified by low-field MRI, with 5 cases (4−10 mm in diameter) only detectable on high-field systems. Larger stroke volumes on 0.064 T MRI were further correlated with poor functional outcomes (T2-weighted infarct volume: good: 7.74 [2.85–22.74] cm³; poor: 64.56 [14.68–170.63] cm³, *p* < 0.01). In another study, Mazurek et al examined intracerebral hemorrhage in 56 patients, finding an 80.4% sensitivity (95% CI: [0.68–0.90]) for hemorrhage detection and 96.6% specificity (95% CI: [0.90–0.99]) for blood-negative cases [63]. Hematoma volumes showed strong correlation with conventional MRI volumes (ICC = 0.955, *p* = 1.69e-30; ICC = 0.875, *p* = 1.66e-8). Finally, Sheth et al assessed midline shift in brain injury patients with a 0.064-T MRI, finding excellent agreement with standard-of-care computed tomography (ICC = 0.94), with sensitivity of 0.93 and specificity of 0.96 [64]. Examples of 0.064-T acquired images compared to 3.0-T images in cases of hydrocephalus, mastoiditis, stroke, and intracranial hemorrhage are provided in Figs. 3 and 4.

## Challenges and limitations

Although lower-field MRI systems offer many advantages—such as lower cost, portability, and safety—they still face several challenges that must be addressed to ensure broader clinical adoption.

### Image quality and SNR

The SNR in MRI is directly proportional to the magnetic field strength and increases with the square root of acquisition time [65]. As such, lower-field systems inherently produce images with lower SNR, requiring longer scan times or advanced techniques to compensate. Simultaneous multislice acquisitions are employed in both high- and low-field MRI systems to accelerate imaging, with variable success. While AI-based reconstruction methods and denoising algorithms have narrowed the image quality gap between low- and high-field systems, these technologies are not without limitations. For example, acceleration-related artifacts—such as fluid signal loss on T2-weighted sequences—have been reported and may be mitigated by disabling simultaneous multislice acquisition in certain protocols [66]. DWI at low-field, particularly on portable scanners, has also shown variable quality due to susceptibility to electromagnetic interference and hardware limitations. In a study by Prabhat et al, only 27% of low-field DWI scans were deemed diagnostically adequate [67]. However, recent software and hardware improvements, along with AI-enhanced reconstructions, have led to markedly improved image quality in newer iterations of these systems [63].

### Fat suppression

Fat suppression based on chemical shift techniques becomes more challenging at lower magnetic field strengths due to the decreased frequency difference between fat and water signals. At 1.5 T, this difference is approximately 220 Hz, whereas at 0.55 T it drops to only around 80 Hz. The smaller spectral separation increases the risk of water-fat signal overlap and makes fat suppression more susceptible to *B*_0_ inhomogeneities and RF pulse imperfections. Early generations of low-field MRI systems struggled with inconsistent fat suppression [68]. However, newer systems have shown significant improvements in uniformity and reliability, largely due to advancements in RF coil design and pulse sequence optimization [40]. Alternative fat suppression techniques, such as the Dixon method, offer improved robustness at low fields. Dixon-based imaging uses a set of in-phase and opposed-phase acquisitions to mathematically separate water and fat signals. This approach is compatible with turbo spin-echo and 3D gradient-echo sequences. When used with T1 weighting, water-only Dixon images can effectively substitute for traditional fat-suppressed T1-weighted scans.

### Limited applications

While low-field MRI systems have demonstrated utility in a variety of clinical areas—especially musculoskeletal and neurological imaging—certain applications remain limited or infeasible at lower field strengths.

Cardiac imaging, for instance, has been shown to be possible at 0.55 T. However, ultra-low-field strengths (< 0.1 T) present greater challenges, such as lower SNR limiting resolution, reduced temporal resolution, and poor spectral fat separation. Additionally, distortion and degradation of electrocardiogram signals impair gating and motion correction, requiring other synchronization techniques such as free breathing.

A prospective study by Zandwijk et al on ten patients after endovascular aneurysm repair imaged on a tiltable 0.25-T low-field system compared results with computed tomography for endoleak detection [69]. Only a 50% concordance was found between low-field MRI and computed tomography. The findings imply that current upright low-field MRI offers limited added value for endoleak evaluation and lacks the spatial fidelity needed for clinically reliable deformation measurements.

Nonetheless, ongoing improvements in acquisition strategies and post-processing may expand the feasible use cases in the future.

## Outlook

Several low-field MRI prototypes and non-FDA-approved systems are currently under development or awaiting regulatory clearance. Ongoing advancements in AI, software optimization, and magnet design continue to drive improvements in image quality and portability, bringing these systems closer to routine clinical use.

### Increasing portability while reducing costs

Halbach arrays—successfully employed in magnetic levitation railways, electric motors, and particle accelerators—are now being adapted for MRI magnet design. These arrays use strategically arranged permanent magnets to produce a strong and uniform *B*_0_ field on one side, while nearly canceling it on the opposite side. This configuration provides three primary benefits: self-shielding, eliminating the need for expensive passive or active shielding; reduced weight, improving portability; and lower manufacturing cost, due to the use of permanent magnets [6].

**Fig. 5:**
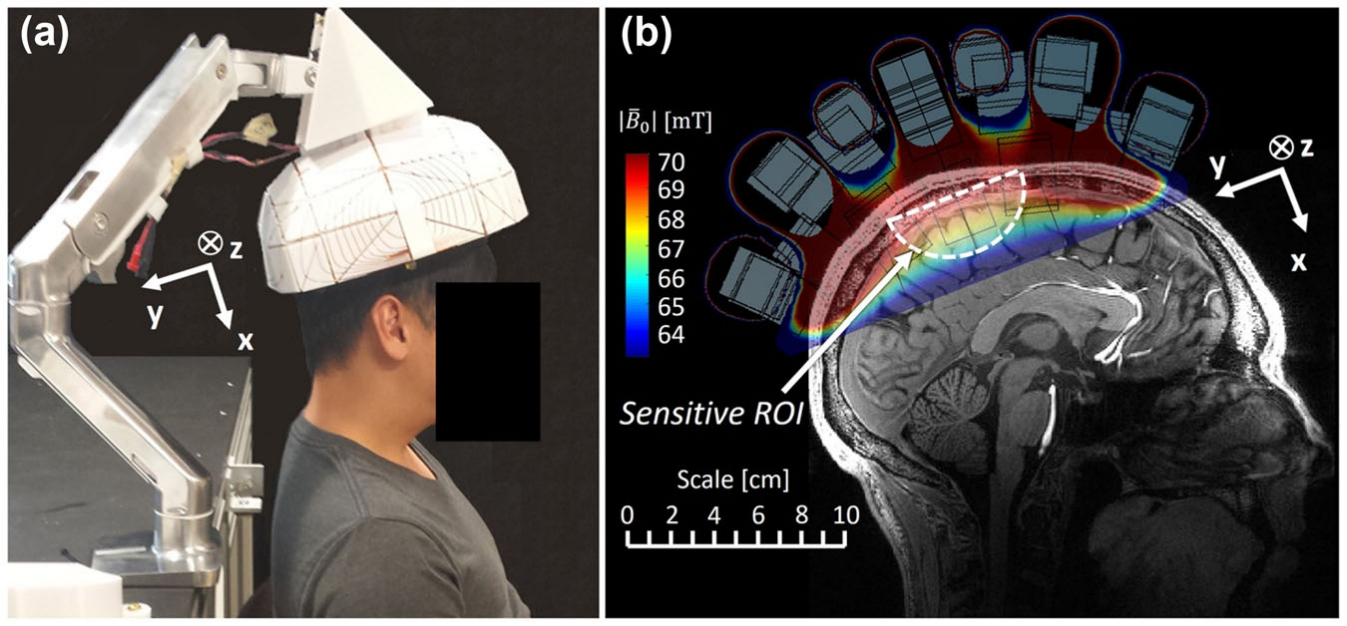
Experimental Halbach arrays used in the “MR” cap illustrating system positioning on a subject’s head (**a**). **b** Shows a proposed *B*_0_ map with a built-in readout encoding gradient. Reprinted with permission and courtesy of McDaniel et al and Wiley Publishing [71]

Phantom studies using Halbach-based systems weighing around 75 kg and constructed for under €10,000 have demonstrated promising T1 and T2 tissue contrast [35]. In Japan, researchers developed a 0.2-T low-field system specifically for joint imaging, mounted inside a minivan to deliver imaging services to underserved rural areas [70]. Pushing the boundaries of portability even further, McDaniel et al developed a wearable Halbach array-based magnetic resonance “cap” for ultra-low-field neuroimaging. Weighing only 8.3 kg, the system achieves a field strength of 0.064 T, with a total cost under $450 USD (Fig. 5) [71]. It is capable of both one dimensional depth profiling and 3D imaging with an in-plane resolution of 2 mm, 6-mm slice thickness, and a scan time of approximately 11 min. In a subsequent project, the team introduced a 0.08-T, 122-kg Halbach-based scanner that required no external power or cooling—offering fully self-contained operation [72]. These innovations lay the groundwork for true point-of-care MRI, enabling imaging in environments such as ambulances, emergency departments, intensive care units, and rural clinics.

### Image reconstruction techniques, integration of automation and AI

Computational advances have played a central role in elevating low-field MRI performance. Fundamentally, MRI trades time for SNR—low-field systems inherently acquire less signal per unit time. To counteract this, modern systems utilize compressed sensing and parallel imaging, both of which have become standard tools to accelerate data acquisition and improve image reconstruction [73]. More recently, the integration of AI and, particularly, DL techniques has revolutionized low-field imaging. AI-driven approaches offer robust capabilities for faster image reconstruction, noise and artifact reduction, and enhanced SNR [74, 75]. These techniques not only compensate for the physical limitations of low-field systems but also create new opportunities for real-time imaging, improved diagnostic confidence, and expanded clinical utility. AI-based algorithms have demonstrated the ability to enable autonomous scan execution and analysis. For instance, a study in Alzheimer’s research used AI to automatically quantify brain morphology and track disease-related changes, illustrating the potential for automated, scalable imaging in both clinical and research settings [76].

### Expansion of clinical applications

Areas of active development include cardiac imaging and intraoperative guidance, where the unique advantages of low-field MRI, such as reduced susceptibility artifacts, enhanced safety for implantable devices, and improved portability, may offer transformative clinical benefits. For example, studies using 0.55-T systems have demonstrated comparable volumetric measurements and strain analysis for cardiac imaging relative to 1.5-T scanners, with excellent correlation for ventricular function and myocardial infarction detection [48–51]. In intraoperative settings, the compact design and low infrastructure requirements of systems like the Synaptive Evry have enabled integration into neurosurgical workflows, resulting in reduced MRI-to-treatment times and improved logistical efficiency [54]. These emerging applications highlight the growing versatility of low-field MRI beyond traditional diagnostic roles.

## Conclusions

We provided a practical overview of the current state of low-field MRI technology. Portable and low-field MRI systems represent a paradigm shift in medical imaging, offering opportunities to overcome traditional barriers posed by high-field MRI systems. These technologies have the potential to expand access to diagnostic imaging in a wide variety of settings—from rural clinics and emergency departments to intensive care units and mobile units. Although limitations remain—including lower SNR, limited application in certain subspecialties, and slower scan times—the trajectory of innovation suggests a promising future. Continued development in hardware, software, and AI will likely accelerate the adoption of these systems and expand their clinical utility.

Currently, low-field MRI serves as a valuable adjunct to standard high-field imaging, particularly for specific use cases. However, with further refinement, these systems may become viable stand-alone tools across many clinical domains. In addition to enhancing diagnostic access, low-field MRI also presents significant economic and environmental advantages for healthcare providers, patients, and health systems worldwide.

## Data Availability

Not applicable.

## Abbreviations

3D: Three-dimensional
AI: Artificial intelligence
CI: Confidence interval
DL: Deep learning
DWI: Diffusion-weighted imaging
FDA: Food and Drug Administration
ICC: Intraclass correlation coefficient
MRI: Magnetic resonance imaging
RF: Radiofrequency
SAR: Specific absorption rate
SNR: Signal-to-noise ratio
